# Selective versus routine preoperative testing in minor gynecologic surgical procedures: A two-center comparative cohort study of perioperative outcomes and resource utilization

**DOI:** 10.1016/j.eurox.2026.100487

**Published:** 2026-08-25

**Authors:** Stefano Restaino, Alice Poli, Martina Arcieri, Federico Paparcura, Giulia Pellecchia, Silvia Zermano, Annalisa Graziano, Chiara Paglietti, Emma Bonetti, Federica Bernardini, Eleonora La Fera, Federica Campolo, Valeria Masciullo, Gaetano Draisci, Stefano Catarci, Teresa Dogareschi, Anna Biasioli, Tiziana Bove, Lorenza Driul, Francesco Fanfani, Giuseppe Vizzielli, Ursula Catena

**Affiliations:** a Clinic of Obstetrics and Gynecology, "Santa Maria della Misericordia" University Hospital, Azienda Sanitaria Universitaria Friuli Centrale, Udine, Italy; b PhD School in Biomedical Sciences, Gender Medicine, Child and Women Health, University of Sassari, Sassari, 07100, Italy; c Department of Medicine, University of Udine, Udine, Italy; d Gynecology and Obstetrics Unit, Policlinico of Abano Terme, Padua, Italy; e S.C. Ostetricia e Ginecologia, ASST Spedali Civili Brescia, Dipartimento Area della Donna e Materno Infantile, Brescia, Italy; f Dipartimento per le Scienze Della Salute Della Donna, del Bambino e di Sanità Pubblica, UOC Ginecologia Oncologica, Fondazione Policlinico Universitario Agostino Gemelli IRCCS, Rome, Italy; g Department of Anesthesia, Emergency and Intensive Care Medicine, Fondazione Policlinico Universitario A. Gemelli IRCCS, Rome, Italy; h Department of Anesthesia and Intensive Care Medicine, ASUFC University Hospital of Udine, Udine, Italy; i Università Cattolica del Sacro Cuore, Rome, Italy

**Keywords:** Minor gynaecologic surgery, Preoperative test, Intraoperative complications, Postoperative complications, Costs

## Abstract

**Background:**

Routine preoperative testing remains common in minor gynaecological surgery, offering opportunities to reduce costs by avoiding unnecessary tests.

**Objectives:**

To compare the clinical safety and cost impact of selective versus routine preoperative laboratory testing in women undergoing minor gynecologic surgical procedures.

**Methods:**

Retrospective two-center comparative cohort study in two tertiary gynecologic centers adopting different preoperative testing policies. A total of 5240 consecutive women undergoing elective minor gynecologic surgical procedures for benign gynecologic pathology were included. Patients were managed according to two different preoperative strategies: routine laboratory testing (Roma cohort) and selective testing based on clinical assessment and comorbidity (Udine cohort). The study aims to compare perioperative outcomes and healthcare costs between routine and selective preoperative testing strategies.

**Outcomes:**

Intraoperative and postoperative complications, healthcare costs in two groups.

**Results:**

Postoperative complications were infrequent in both cohorts and did not differ significantly between routine and selective testing strategies (p = 0.345). In multivariable logistic regression analysis, selective testing was not independently associated with postoperative complications (p = 0.345). Independent predictors of complications included age (p = 0.041), ASA score ≥ 3 (p = 0.021), and operative time (p = 0.050). Selective testing was associated with a significant reduction in preoperative laboratory costs without negatively affecting perioperative safety.

**Conclusion:**

In minor gynecologic surgical procedures, selective preoperative laboratory testing based on clinical evaluation appears to be as safe as routine testing while reducing healthcare expenditures.

**What is new?:**

A more tailored and guideline-concordant preoperative assessment strategy in minor gynecologic surgery may reduce healthcare expenditure.

## 1 Introduction

Minor gynaecological surgery includes a range of minimally invasive procedures, mainly hysteroscopy and hysteroscopy-guided interventions such as endometrial biopsy, intrauterine device (IUD) insertion, polypectomy, myomectomy, metroplasty, and isthmoplasty. Other procedures include loop electrosurgical excision procedure (LEEP), cervical conization, excision of vulvar lesions, and blind dilation and curettage (D&C). These interventions are generally performed in outpatient or day-surgery settings and are widely used because of the high prevalence of intrauterine pathology and abnormal uterine bleeding [1].

Evidence consistently shows that minor gynaecological surgery is associated with very low complication rates. A multicentre study of 13,600 procedures reported complication rates of 0.13% for diagnostic hysteroscopy and 0.28% for operative hysteroscopy, with most events related to uterine cavity entry or surgical experience [2]. Similarly, Kayatas et al. analysed 5474 hysteroscopies and found uterine perforation to be the most common complication (0.27%), with rates of 0.06% and 1% for diagnostic and operative procedures, respectively [3]. An interim analysis of the present study confirmed these findings, reporting postoperative complication rates of 1.2–1.4% and severe complications in only 0.3–0.4% of cases [4].

Many centres routinely perform a preoperative assessment consisting of medical history, physical examination, review of previous records, and ultrasonography. Additional investigations may include blood tests, urinalysis, ECG, chest X-ray, or further examinations required for anaesthetic evaluation. While these assessments aim to improve patient safety and identify individuals requiring perioperative optimisation, they also contribute substantially to healthcare expenditure [5].

Richman et al. estimated that approximately $18 billion is spent annually on preoperative testing in the United States [6]. Ferrando et al. demonstrated that adherence to guideline-based testing could reduce per-patient costs by 63% [7]. These findings highlight the importance of evaluating whether routine testing is necessary in low-risk patients.

Current guidelines from the American Society of Anesthesiologists (ASA) recommend against routine preoperative testing in the absence of clinical indications. Instead, assessment should be based on medical history, physical examination, and existing documentation, with further investigations reserved for cases in which results may influence perioperative management [8]. Similar recommendations have been issued by the European Society of Anaesthesiology and Intensive Care (ESAIC), NICE, and the ACC/AHA [[9], [10], [11]].

This study aims to confirm findings from a 2024 interim analysis by assessing whether preoperative testing, in the absence of risk factors, affects perioperative outcomes in patients undergoing minor gynaecological surgery and by evaluating the cost-effectiveness of different preoperative assessment strategies.

## 2 Material and methods

### 2.1 Study design and setting

This was a multicenter, observational, comparative cohort study conducted at two tertiary gynecologic referral centers with different institutional approaches to preoperative laboratory testing. The study was designed and reported in accordance with STROBE recommendations for observational studies.

During the study period, patients were managed according to the standard preoperative protocol at each institution. At Policlinico Universitario Fondazione Gemelli IRCCS (Rome), routine preoperative laboratory testing and a chest X-ray were performed before elective minor gynecologic surgery. At Azienda Sanitaria Universitaria Friuli Centrale (Udine), laboratory investigations were performed only when clinically indicated after medical history and physical examination, in line with current guideline recommendations [[8], [9], [10], [11]].

### 2.2 Study population

We retrospectively analyzed consecutive adult women undergoing elective minor gynecologic surgery during the predefined study interval (January 2017 and December 2023). Minor procedures included diagnostic and operative hysteroscopy (endometrial biopsy, IUD insertion, polypectomy, myomectomy, metroplasty, isthmoplasty), loop electrosurgical excision procedure (LEEP), cervical conization, excision of vulvar lesions, and blind dilation and curettage (D&C), consistent with the international terminology consensus [1].

Emergency procedures and cases with incomplete perioperative data were excluded.

Cases with incomplete key perioperative data were also excluded from the final analysis.

Clinical practice and procedural/documentation practices remained unchanged at both centers during the COVID-19 period, the only additional requirement was a negative nasopharyngeal swab.

### 2.3 Exposure definition

The exposure variable was the institutional preoperative testing strategy:

• Group A (Rome): routine preoperative blood testing and chest X-ray.
• Group B (Udine): selective testing based on clinical indication (ASA≥3, immunosuppression, known electrolyte abnormalities, known difficult airways, previous major adverse reaction to anesthesia, history of abnormal bleeding, heart failure or significant arrhythmias).

Laboratory testing in Group A typically included complete blood count, coagulation profile, renal function tests, and other standard panels, per institutional policy.

This exposure was defined at the institutional level rather than the individual level, reflecting real-world policy-driven practice patterns.

### 2.4 Outcomes

The primary outcome was the occurrence of perioperative complications.

Secondary outcomes included:

• Intraoperative complications
• Postoperative complications within 30 days
• Surgical reintervention
• Hospital readmission
• Direct testing-related costs

Complications were graded according to the Clavien–Dindo classification.

### 2.5 Data collection

Demographic and clinical variables included age, BMI, ASA score, type of procedure, operative time, and hospital stay.

Our institutional protocol included standardized baseline questions, often also administered by the anesthesiologist, which were routinely used to identify relevant medical conditions that might not have been adequately reported by patients, particularly those with limited health literacy or difficulty in accurately reporting their medical history. This approach helped to ensure that patients with potentially relevant comorbidities were appropriately identified and, when necessary, referred for further preoperative assessment. When patients are unable to provide a complete or reliable medical history, we review our institutional electronic medical records to identify previous hospital admissions, diagnoses, or relevant medical conditions; when appropriate, we seek additional information from family members or caregivers and ask patients to provide available medical records or documentation concerning previous health problems. In selected cases, when clinically relevant information remains unavailable or unclear, we also contact the patient’s general practitioner, who may provide further information regarding the patient’s medical history and chronic conditions.

Approval from the Institutional Review Board (IRB) of University of Udine was obtained on February 7th, 2022 (approval code n. 40/2022). An amendment to the original IRB approval was subsequently obtained to extend the study period through December 2023.

### 2.6 Statistical analysis

Continuous variables were expressed as median (range) and compared using the Mann–Whitney *U* test. Categorical variables were expressed as frequencies and percentages and compared using chi-square or Fisher’s exact test, as appropriate. Multivariable logistic regression analysis was performed to identify independent predictors of perioperative complications (Clavien–Dindo grade >0). Variables included in the model were selected based on clinical relevance and univariable screening. Adjusted odds ratios (aOR) with 95% confidence intervals (CI) were calculated. Given the low complication rate, the model was kept parsimonious to avoid overfitting. Statistical significance was set at p < 0.05. Analyses were performed using NCSS 11 Statistical Software (2016). NCSS, LLC. Kaysville, Utah, USA, ncss.com/software/ncss.

## 3 Results

A total of 5240 patients were included: 2887 in Group A (routine testing) and 2353 in Group B (selective testing). Within Group B, preoperative testing was performed on 47 patients (2% of the cohort) when clinically indicated based on the previously reported medical conditions.

Baseline characteristics are summarized in Table 1.

**Table 1:** Patient’s characteristics.

	**Group A (Rome)**	**Group B (Udine)**	**p value**
Number of patients	2887	2353	-
Median age (range)	49.3 (14−93)	53.8 (12−99)	< 0.0001
Median BMI Kg/m2 (range)	25.1 (15−56)	25.5 (15−61)	0.01
Median ASA (range)	2 (1−3)	2 (1−3)	1

Median age was slightly higher in Group B (53.8 years vs 49.3 years; p < 0.0001). Median BMI differed modestly between groups (25.5 vs 25.1 kg/m²; p = 0.01). Median ASA score was 2 in both groups, indicating comparable overall preoperative risk.

### 3.1 Surgical procedures

The distribution of procedures differed between centers (Table 2), reflecting institutional specialization. More complex hysteroscopic procedures (e.g., myomectomy, metroplasty) were more frequently performed in Rome, whereas blind D&C and conization were more common in Udine.

**Table 2:** Type of surgical procedures performed in each center.

	**Centre A (Rome) N (%)**	**Centre B (Udine) N (%)**
**Type of surgery** Diagnostic hysteroscopy + endometrial biopsy Diagnostic hysteroscopy + IUD insertion Polypectomy Myomectomy Blind D&C LEEP/conization Vulvar procedures Metroplasty/isthmoplasty	Tot 2887 444 (15.4%) 15 (0.5%) 1717 (59.5%) 371 (12.8%) 61 (2.1%) 124 (4.3%) 49 (1.7%) 106 (3.7%)	Tot 2353 575 (24.4%) 5 (0.2%) 689 (29.3%) 78 (3.3%) 575 (24.4%) 319 (13.6%) 66 (2.8%) 46 (2%)

### 3.2 Perioperative outcomes

Perioperative outcomes are detailed in Table 3.

**Table 3:** Surgical features, perioperative outcomes and costs.

	**Group A (Rome)**	**Group B (Udine)**	**p value**
All patients – N°	2887	2353	-
Median surgical time (range); minutes	19 (2−115)	18 (2−90)	0.005
Median hospital stay (range); days	0 (0−10)	0 (0−10)	1
Surgical reintervention – N° (%)	4 (0.14%)	6 (0.25%)	0.36
Surgical complications – N° (%) Intraoperative complications – N° Postoperative complications – N° Clavien-Dindo grade I Clavien-Dindo grade II Clavien-Dindo grade III	22 (0.8%) 11 (0.4%) 11 (0.4%) 10 5 7	28 (1.2%) 8 (0.3%) 20 (0.9%) 13 6 9	0.12 - - - - -
Severe complications – N° (%)	7 (0.2%)	9 (0.4%)	0.45
Lab test costs for each procedure; €	55	0	< 0.001
Chest X-Ray costs for each procedure; €	15	0	< 0.001

Overall complication rates were low in both groups, confirming the intrinsic safety profile of minor gynecologic surgery. In Group A (routine testing), complications occurred in 0.8% of cases, compared with 1.2% in Group B (selective testing), with no statistically significant difference (p = 0.12).

Intraoperative complications were rare and mainly consisted of uterine perforation and fluid overload during hysteroscopic procedures. Postoperative complications included metrorrhagia, transient anemia, abdominal pain, and suspected infections. Severe complications (Clavien–Dindo grade ≥III) were uncommon in both cohorts (0.2% vs 0.4%, p = 0.45), and no grade IV or V events were recorded. Importantly, among patients who underwent preoperative laboratory testing, no major complication appeared to be preventable through routine laboratory screening. In particular, patients who developed postoperative bleeding had no abnormal preoperative coagulation parameters.

Median operative time was slightly longer in Group A (19 vs 18 min; p = 0.005), although the difference was not clinically meaningful. Median length of hospital stay was identical in both groups (0 days; p = 1), reflecting the predominantly day-surgery nature of the procedures.

Rates of surgical reintervention (0.14% vs 0.25%; p = 0.36) and hospital readmission were similarly low and not significantly different between the two strategies.

Taken together, these findings suggest that routine preoperative laboratory testing did not translate into measurable improvements in perioperative safety.

### 3.3 Cost analysis

**Fig. 1:**
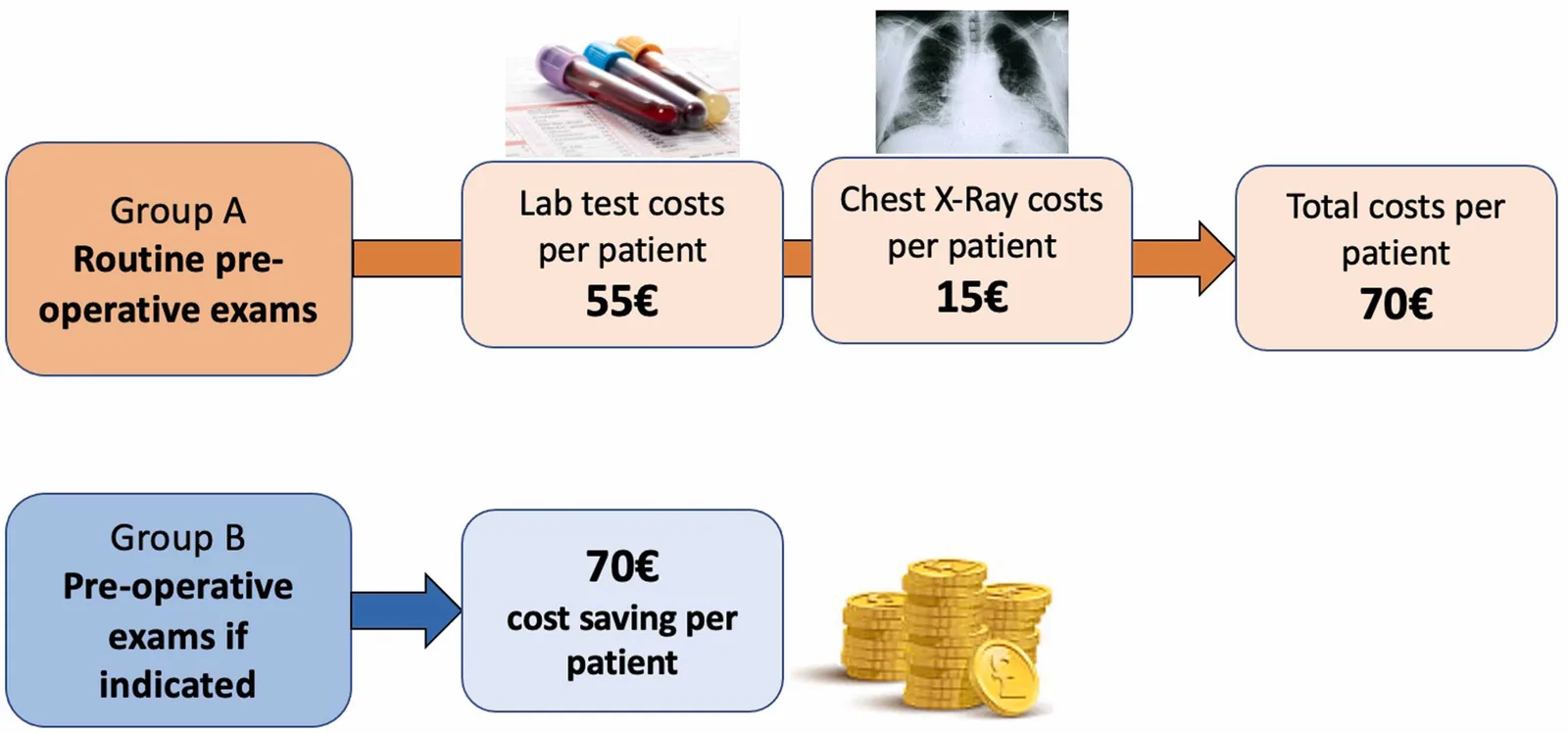
Illustrative comparison of cost differences between group A and group B approaches.

Regarding costs, Table 3 and Fig. 1 illustrate the economic impact of the two preoperative assessment strategies. In the routine testing group, the direct cost of laboratory tests (€55) and chest X-ray (€15) resulted in an additional fixed expenditure of approximately €70 per patient. In contrast, in the selective testing group, these investigations were performed only when clinically indicated, resulting in a statistically significant reduction in direct preoperative costs (p < 0.001).

Fig. 1 illustrates the cost differential between the two institutional approaches. When extrapolated to the high annual volume of minor gynecological procedures, this difference translates into a substantial cumulative financial impact on the healthcare system. Importantly, this cost reduction was not associated with an increase in perioperative complications, readmissions, or reinterventions, reinforcing the cost-effectiveness of a selective, guideline-based testing strategy.

### 3.4 Multivariable analysis

A multivariable logistic regression analysis was performed to identify factors independently associated with postoperative complications. Variables were selected based on clinical relevance and univariable screening, and included age, BMI, ASA class, operative time, and preoperative testing strategy (routine vs selective), as detailed in the Methods. As shown in Table 4, the preoperative testing strategy was not independently associated with postoperative complications after adjustment for potential confounders (adjusted OR 0.73, 95% CI 0.39–1.40; p = 0.345). In contrast, patient-related risk (e.g., higher ASA class) and procedural factors (e.g., longer operative time) were more strongly associated with the occurrence of complications. Age showed a modest association with the risk of complications, whereas BMI did not retain independent significance in the adjusted model. Overall, these findings suggest that perioperative morbidity in minor gynecologic surgery is driven primarily by baseline clinical status and procedural characteristics rather than routine preoperative laboratory screening.

**Table 4:** Multivariable logistic regression for perioperative complications (Grade > 0, n = 41).

**Predictor**	**Adjusted OR**	**95% CI**	**p-value**
Group A (Rome) Group B (Udine)	0.73	0.39 – 1.40	0.345
Age (per 1-year increase)	1.02	1.00 – 1.05	**0.041**
BMI (per 1 kg/m² increase)	0.98	0.93 – 1.03	0.330
ASA ≥ 3 ASA 1–2	3.24	1.19 – 8.83	**0.021**
Operative time (per 30-min increase)	1.80	1.00 – 3.23	0.050

## 4 Discussion

### 4.1 Main findings

In this two-center retrospective comparative study including more than 5000 hysteroscopic procedures, we found that selective preoperative laboratory testing based on clinical evaluation was not associated with an increased risk of postoperative complications compared with routine testing. Complication rates were low in both cohorts, consistent with the well-established safety profile of hysteroscopic surgery in appropriately selected patients [[2], [3], [12], [13], [14]]. Importantly, after adjustment for potential confounders, the preoperative testing strategy was not independently associated with adverse outcomes, whereas patient-related factors (age and ASA score) and procedural complexity (operative time) were significant predictors.

These findings align with current international recommendations discouraging routine laboratory testing before low-risk surgical procedures in otherwise healthy patients [[5], [6], [7], [8], [9], [10], [11], [12], [15]]. The American Society of Anesthesiologists, NICE, and the European Society of Anaesthesiology and Intensive Care all emphasize that preoperative investigations should be individualized and guided by clinical history and physical examination rather than applied systematically [[8], [9], [10], [11]]. Our data provide real-world surgical evidence supporting this approach specifically in hysteroscopic surgery, an area where routine testing is still frequently performed despite limited supporting evidence.

The absence of benefit from routine laboratory testing is biologically plausible. Most hysteroscopic procedures are short, minimally invasive, and associated with minimal blood loss and low anesthetic risk [[1], [2], [16]]. In this context, abnormal laboratory findings rarely modify perioperative management in asymptomatic ASA I–II patients [[6], [12]]. Instead, our multivariable analysis confirmed that global clinical status (ASA ≥3) and increasing age are more relevant determinants of postoperative complications than laboratory screening per se. Similarly, operative time — a surrogate of procedural complexity — was associated with borderline statistical significance, reinforcing that surgical factors may outweigh routine biochemical assessment in determining perioperative risk.

From a health system perspective, the cost analysis demonstrated a significant reduction in preoperative expenditures with a selective testing strategy, without compromising safety. Although hysteroscopy is typically performed in ambulatory settings, cumulative unnecessary testing may generate substantial economic burden [[7], [12]]. In value-based healthcare systems — including those with mixed public-private reimbursement models — eliminating low-yield investigations represents a rational strategy to improve efficiency while preserving quality of care.

### 4.2 Strengths and limitations

Several limitations must be acknowledged. First, the retrospective design introduces the possibility of selection bias and unmeasured confounding. However, the inclusion of consecutive patients and the adjustment for major clinical covariates strengthen the internal validity of the analysis. Second, although the overall sample size was large, complication rates were low, which may limit the statistical power to detect small differences. Third, indirect societal costs were not assessed, and our cost analysis was limited to direct preoperative laboratory expenses. Finally, another limitation is the different distribution of procedure types across centers, which may have introduced a potential source of bias.

Despite these limitations, the study has important strengths. The large cohort size, real-world multicenter setting, and multivariable risk adjustment provide robust clinical evidence supporting a selective approach. To our knowledge, this represents one of the largest comparative analyses specifically evaluating preoperative testing strategies in minor gynecologic surgery.

### 4.3 Strengths and limitations compared to other studies

Notably, prior studies have primarily focused on anesthesiology or general surgical populations [[6], [8], [9]], whereas minor gynecologic surgery has been less extensively addressed in large contemporary cohorts.

### 4.4 Clinical and policy implications

Our findings are particularly relevant given the increasing demand for minor gynecologic surgical procedures and outpatient surgery [[5], [17]]. Standardization of evidence-based preoperative pathways may contribute to resource optimization and reduce variability in clinical practice.

## 5 Conclusions

Selective preoperative laboratory testing based on individualized clinical assessment may be a reasonable approach in women undergoing minor gynecologic surgical procedures. In our cohort, routine laboratory screening was not associated with a reduction in postoperative complications, while it may contribute to additional healthcare expenditure in appropriately selected patients. Further prospective studies are warranted to validate these findings and to assess whether standardized, risk-based preoperative pathways could safely optimize laboratory testing in minor gynecologic surgical procedures.

## CRediT authorship contribution statement

**Stefano Restaino:** Writing – review & editing, Writing – original draft, Visualization, Validation, Methodology, Formal analysis, Conceptualization. **Alice Poli:** Writing – original draft, Resources, Methodology, Investigation, Formal analysis, Data curation. **Martina Arcieri:** Writing – review & editing, Methodology, Investigation. **Federico Paparcura:** Data curation. **Giulia Pellecchia:** Data curation. **Silvia Zermano:** Data curation. **Annalisa Graziano:** Data curation. **Chiara Paglietti:** Data curation. **Emma Bonetti:** Supervision. **Federica Bernardini:** Supervision. **Eleonora La Fera:** Supervision. **Federica Campolo:** Supervision. **Valeria Masciullo:** Supervision. **Gaetano Draisci:** Supervision. **Stefano Catarci:** Supervision. **Teresa Dogareschi:** Supervision. **Anna Biasioli:** Supervision. **Tiziana Bove:** Supervision. **Lorenza Driul:** Validation, Project administration. **Francesco Fanfani:** Validation, Project administration. **Giuseppe Vizzielli:** Writing – review & editing, Validation, Project administration, Conceptualization. **Ursula Catena:** Writing – review & editing, Methodology, Conceptualization.

## Ethical approval

Approval from the Institutional Review Board of University of Udine was obtained on February 7th, 2022 (approval code n. 40/2022).

## Informed consent

Informed consent was waived due to the retrospective nature of the study and the use of de-identified data.

## Funding

This research did not receive any specific grant from funding agencies in the public, commercial, or not-for-profit sectors.

## Data sharing

The datasets analyzed during the current study are available from the corresponding author on reasonable request.

## Transparency

The authors affirm that this manuscript is an honest, accurate, and transparent account of the study being reported; and that no important aspects of the study have been omitted.

## Declaration of Competing Interest

The authors declare that they have no known competing financial interests or personal relationships that could have appeared to influence the work reported in this paper
